# Consistent Biomarkers and Related Pathogenesis Underlying Asthma Revealed by Systems Biology Approach

**DOI:** 10.3390/ijms20164037

**Published:** 2019-08-19

**Authors:** Xiner Nie, Jinyi Wei, Youjin Hao, Jingxin Tao, Yinghong Li, Mingwei Liu, Boying Xu, Bo Li

**Affiliations:** 1 College of Life Sciences, Chongqing Normal University, Chongqing 401331, China; 2 School of Biological Information, Chongqing University of Posts and Telecommunications, Chongqing 400065, China; 3 College of Laboratory Medicine, Chongqing Medical University, Chongqing 400046, China

**Keywords:** Asthma, Epithelial cells, Data integrating, Gene set enrichment analysis, Biomarker, Protein-protein interaction network.

## Abstract

Asthma is a common chronic airway disease worldwide. Due to its clinical and genetic heterogeneity, the cellular and molecular processes in asthma are highly complex and relatively unknown. To discover novel biomarkers and the molecular mechanisms underlying asthma, several studies have been conducted by focusing on gene expression patterns in epithelium through microarray analysis. However, few robust specific biomarkers were identified and some inconsistent results were observed. Therefore, it is imperative to conduct a robust analysis to solve these problems. Herein, an integrated gene expression analysis of ten independent, publicly available microarray data of bronchial epithelial cells from 348 asthmatic patients and 208 healthy controls was performed. As a result, 78 up- and 75 down-regulated genes were identified in bronchial epithelium of asthmatics. Comprehensive functional enrichment and pathway analysis revealed that response to chemical stimulus, extracellular region, pathways in cancer, and arachidonic acid metabolism were the four most significantly enriched terms. In the protein-protein interaction network, three main communities associated with cytoskeleton, response to lipid, and regulation of response to stimulus were established, and the most highly ranked 6 hub genes (up-regulated *CD44, KRT6A*, *CEACAM5*, *SERPINB2*, and down-regulated *LTF* and *MUC5B*) were identified and should be considered as new biomarkers. Pathway cross-talk analysis highlights that signaling pathways mediated by IL-4/13 and transcription factor HIF-1α and FOXA1 play crucial roles in the pathogenesis of asthma. Interestingly, three chemicals, polyphenol catechin, antibiotic lomefloxacin, and natural alkaloid boldine, were predicted and may be potential drugs for asthma treatment. Taken together, our findings shed new light on the common molecular pathogenesis mechanisms of asthma and provide theoretical support for further clinical therapeutic studies.

## 1. Introduction

Asthma is a chronic inflammatory disease of the airways, caused by genetic or environmental and lifestyle factors, which is characterized by airway hyper-responsiveness (AHR), inflammation, and variable airflow obstruction [1]. Despite recent progress in the development of anti-asthmatic medication, asthma is still a major public health problem in the world. Its prevalence, morbidity, and mortality are still increasing [2]. According to the Global Asthma Report 2018, asthma affects over 339 million people, resulting in more than 1000 deaths every day (http://globalasthmareport.org). By 2025, an additional 100 million people may develop asthma [3]. However, this may be an underestimation due to underdiagnosis. A recent national survey in China found that the prevalence rate was ~4.2% in adults over 20, and over 45.7 million people suffered from asthma [4]. The total cost of asthma exceeded ~$81 billion per year in the USA and ~72€ billion in Europe [5]. Therefore, asthma not only affects human health seriously but also leads to serious socio-economic problems.

As a T-lymphocyte-controlled airway disease, asthma is caused by inflammation, overproduction of mucus, and airway remodeling, which results in hyper-reactivity and airway obstruction. The airway epithelial cells act as the first physical and immunological barrier and play an extremely important role in controlling inflammatory, immune, and regenerative responses to microorganism infections, allergens, and environmental pollutants that contribute to asthma pathogenesis [6]. Epithelial cells express pattern recognition receptors that detect the stimuli and release chemokines and cytokines, thereby bridging the innate and adaptive immune system cells [7]. Therefore, a better understanding of the underlying molecular mechanisms of the epithelium’s function in maintaining the integrity of the airways and its dysfunction in asthma is crucial for exploring how asthma is initiated and perpetuated and is also helpful in selecting new biomarkers for guiding therapeutic decision in asthma treatment.

Gene expression analysis is becoming more important in diagnostic fields allowing the identification of novel biomarkers relevant to diseases. The high throughput microarray is an efficient approach that facilitates gene expression analysis, new drug target identification, and novel gene function prediction. Woodruff et al. identified 22 differentially expressed genes (DEGs) (e.g., *CLCA1*, *POSTN*, and *SERPINB1*) in asthmatics when compared to healthy controls [8]. To identify gene expression patterns in children with asthma, Kicic et al. found 1612 genes (including 764 up-regulated and 848 down-regulated genes) were significantly differentially expressed in the epithelium of atopic asthmatics based on microarray analysis. The genes involved in the response to wounding were significantly reduced in epithelial cells, causing deficient immune responses and wound repair [9]. These findings strongly support that a fundamental alteration in the epithelium contributes to the initiation and progression of asthma. To link gene expression profiles to clinical asthma phenotype, Modena et al. performed a microarray analysis for 155 subjects with asthma and healthy controls and identified 1384 differentially expressed genes in bronchial epithelial cells. Further analysis revealed that some genes associated with type 2 inflammation, neuronal function, WNT signaling, and actin cytoskeleton could be considered as potential biomarkers for asthma [10]. A recent study using Affymetrix HT HG-U133+ PM GeneChips found that IL-13 response genes (*POSTN*, *SERPINB2*, *CLCA1*), mast cell mediator genes (*CPA3* and *TPSAB1*), and cystatin genes (*CST1*, *CST2* and *CST4*) were overexpressed in the epithelium of asthmatics [11], which was consistent with their previous study suggesting that activated T cells may be driving neutrophilic inflammation.

Although microarray experiments have generated long lists of genes with altered expression in asthmatics, some inconsistent findings were observed. Therefore, it is necessary to apply a systematic approach to combine different publicly available datasets to explore shared molecular mechanisms with asthma. The powerful meta-analytic technique has been well established to study the shared biological signatures between related diseases and pathophysiological conditions by merging multiple omics datasets [12,13].

In this study, we selected ten eligible microarray datasets to identify genes associated with dysfunctions of the bronchial epithelium in asthmatics. Differentially expressed genes were identified and functional annotations were performed for significant genes by using the microarray data integrating and analyzing techniques. Furthermore, approaches from systems biology, such as enrichment analysis and network analysis, were adopted to look for a better understanding of the molecular mechanisms underlying asthma.

## 2. Results and Discussion

### 2.1. Ten Independent Datasets Meeting the Criteria in This Study

In the study, 191 microarray datasets were obtained by keyword searching (as of 20th December 2018). After filtering based on dataset inclusion and exclusion criteria, 10 asthma-related microarray datasets from six disparate platforms (Affymetrix: HG-U95Av2, HG-U133A, HG-U133_Plus_2, HT_HG-U133_ Plus_PM, and Agilent: 4 × 44K G4112F and SurePrint G3 Human GE v3 8 × 60K) were selected. The detailed information of these datasets and sample descriptions is shown in Table 1 and Supplementary Material S1, respectively.

**Figure 1:**
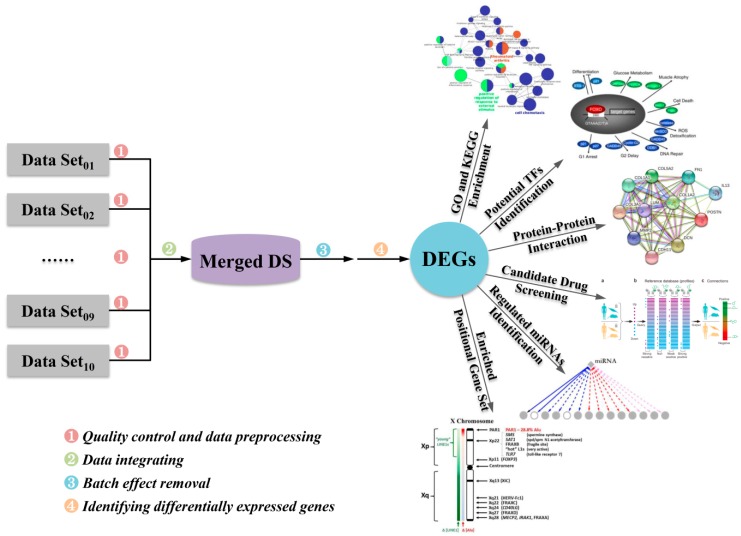
The workflow of microarray data integration and subsequent analyses in this study. Quality control of each dataset was manually checked, and then 10 preprocessed and independent qualifying datasets were merged into a large dataset (designated as Merged DS) for further analysis. Batch effects were removed by using the algorithm of batch effect removal. Differentially expressed genes (DEGs) between the asthmatics and healthy controls were identified based on expression fold change > 1.2 and false discovery rate (FDR) < 0.05. Gene ontology (GO) and Kyoto Encyclopedia of Genes and Genomes (KEGG)-based pathways were subsequently enriched and followed by protein-protein interaction (PPI) network construction, prediction of potential transcription factors (TFs) and microRNAs, chromosomal localization, and potential candidate chemical prediction for asthma treatment. The partial contents in this workflow were cited from published papers [18,19,20,21].

To compile expression data for data integrating and subsequent investigation, each dataset was preprocessed, normalized, and then integrated for further analysis following the steps illustrated in Figure 1.

### 2.2. Large Microarray Dataset Generated from Ten Selective Datasets

#### 2.2.1. Sample Quality Control and Microarray Data Preprocessing

After the quality control, 1 (GSE470), 2 (GSE4302), 2 (GSE18965), 11 (GSE41861), 4 (GSE63142), 3 (GSE64913), 7 (GSE89809), and 4 (GSE104468) samples did not meet the cut-off criteria in array quality metrics assessment and were excluded for subsequent analysis; the QC reports are presented in Supplementary Material S2. Thus, a total of 556 samples were used for further analysis, containing 348 asthmatic patients and 208 healthy subjects. Through microarray data preprocessing, 10 gene expression matrices (genes in rows and samples in columns) were generated. For these matrices, all gene names were replaced by Entrez gene identifiers (IDs).

#### 2.2.2. Data Integration and Batch Correction

**Figure 2:**
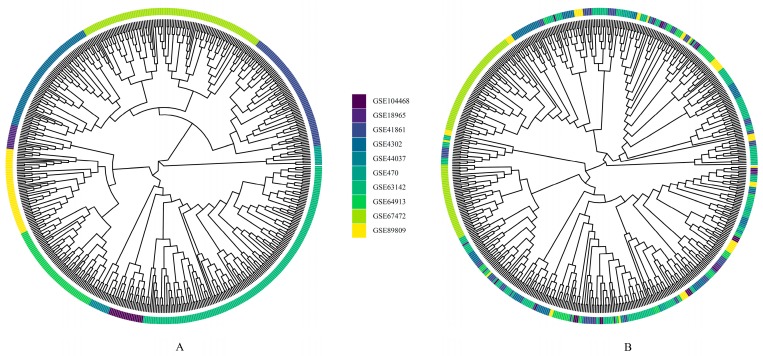
Exploration and visualization of the Merged dataset (abbr. DS) before and after batch correcting. Hierarchical clustering for samples was performed based on *Euclidean* distance and *Complete* linkage. Then, the results of clustering were visualized using clustering dendrograms in ggtree package. (**A**) Before batch effect removal, and (**B**) after batch effect removal.

Based on the results of the data preprocessing, probe annotations, and gene matching, a total of 8324 genes from 556 samples shared by six microarray platforms were selected (Supplementary Figure S1). After data integrating, batch effects were corrected using the ComBat algorithm. The clustering dendrogram showed that each dataset was clearly separated from the others before the batch correction (Figure 2A). After the batch correction, the samples from all datasets were well intermixed (Figure 2B), suggesting that the batch-adjusted data were suitable for further analysis.

### 2.3. Identification of DEGs Involved in Pathogenesis of Asthma

**Figure 3:**
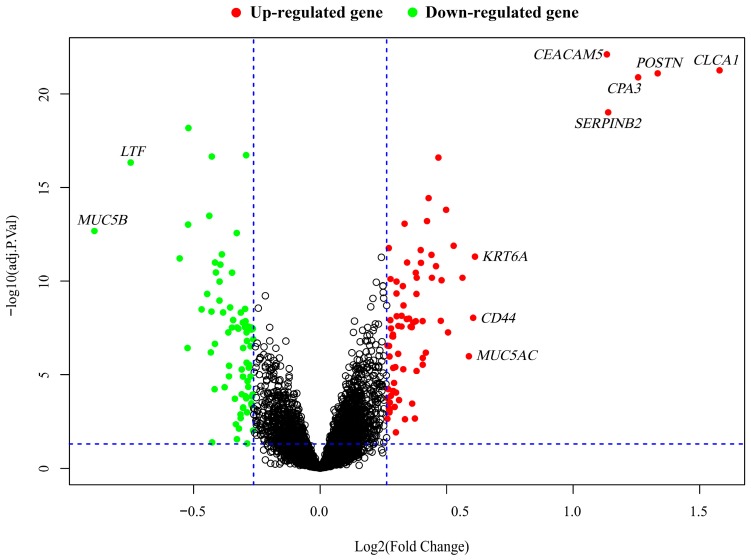
Volcano plot for 153 differentially expressed genes between asthmatics and healthy controls. The up- and down-regulated genes are marked in red and green, respectively. Genes with fold change more than 1.5-fold are labeled by gene symbols.

Based on feature selection, we identified 153 differentially expressed genes including 78 up-regulated and 75 down-regulated genes across the integrated dataset with the fold change > 1.2 and FDR < 0.05 (Table 2 and Supplementary Material S3). To identify genes most closely related to asthma, more stringent criteria (fold change > 1.5 and FDR < 0.05) were applied and only 8 up-regulated, 2 down-regulated genes were obtained. The up-regulated genes included *CEACAM5* (carcinoembryonic antigen), *CLCA1* (calcium-activated chloride channel regulator 1), *POSTN* (periostin), *CPA3* (carboxypeptidase A3), *SERPINB2* (plasminogen activator inhibitor-2), *KRT6A* (keratin 6A), *CD44* (hyaluronate receptor), and *MUC5AC* (mucin 5AC), while *LTF* (lactotransferrin) and *MUC5B* (mucin 5B) were down-regulated. Gene expression patterns of all DEGs are shown in the volcano plot (Figure 3).

### 2.4. Functional Annotations and Enrichment Analysis of DEGs

#### 2.4.1. Gene Ontology Analysis of DEGs

**Figure 4:**
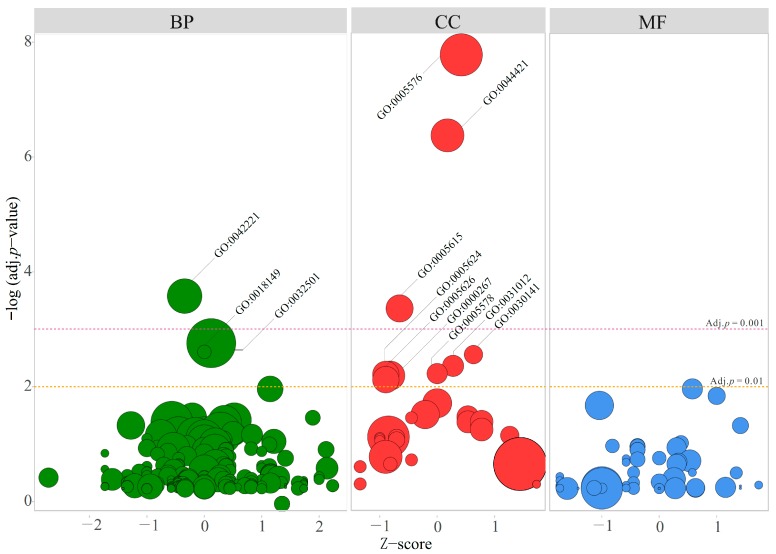
Bubble plot for GO term enrichment. The bubble size represents the number of DEGs in each GO term. Three levels of GO terms are the biological process (BP), cellular component (CC), and molecular function (MF).

Based on DAVID analysis, a total of 12 GO functional terms were enriched with adjusted *p* < 0.01 and a minimum gene count > 6 (Table 3). Among these dysregulated GO terms, response to chemical stimulus (GO:0042221, Z-score = −0.34) was the most significantly enriched into biological process (BP) (Adj. *p* = 2.64 × 10^−4^), while membrane fraction (GO:0005624, Z-score = −0.89), insoluble fraction (GO:0005626, Z-score = −0.89), and cell fraction (GO:0000267, Z-score = −0.82) from cellular components (CC) were considered as the top three decreased terms (Figure 4). Interestingly, two GO terms relevant to asthma were also enriched: increased secretory granule (GO:0030141, Z-score = 0.63) and more active extracellular region (GO:0005576, Z-score = 0.42).

#### 2.4.2. KEGG Pathway Analysis of DEGs

Based on KEGG pathway mapping, DEGs were significantly enriched in 12 pathways (FDR < 0.05) involved in cancer, arachidonic acid metabolism, linoleic acid metabolism, calcium signaling, aldosterone-regulated sodium reabsorption, and so on (Table 4). It is worth noting that 7 down- and 3 up-regulated genes involved in cancer were also enriched (FDR = 3.24 × 10^−5^, Z-score = −1.26), which is consistent with the previous report that asthma status was associated with decreased risk of aggressive bladder cancer [22]. Moreover, the pathway associated with bladder cancer was also enriched (FDR = 1.17 × 10^−2^) with a suppressed trend (Z-score = −0.58). Arachidonic acid metabolism was the second most significant pathway (FDR = 1.42 × 10^−4^). Its abnormal metabolism has been observed in asthma [23,24]. Dysregulated linoleic acid metabolism (FDR = 7.69 × 10^−3^) was the third enriched pathway. Woods et al. proposed that fatty acid levels were associated with the risk of asthma in young adults [25]. Black et al. argued that increasing dietary intake of n-6 linoleic acid had resulted in increased arachidonic acid and PGE2 production, with a consequent increase in the likelihood of asthma [26]. Our results also found the calcium signaling pathway was significantly suppressed in asthma (FDR = 1.17 × 10^−2^, Z-score = −1.34). An abnormal calcium signaling pathway has been linked with many diseases. Mahn et al. reported that the dysregulation of Ca^2+^ homeostasis was probably related to abnormal asthmatic phenotype [27]. Our result supports the fact that peroxisome proliferator-activated receptors (PPAR) are associated with obstructive lung disease [28]. Thus, PPAR may also be a potential target of asthma. Interestingly, a novel pathway named Hematopoietic cell lineage was significantly enriched in this study, suggesting some components in this pathway may be associated with asthma pathogenesis.

#### 2.4.3. Potential Target Sites of Transcription Factors and Regulatory MicroRNAs

Using the GSEA online tool, 9 DNA-binding motifs with FDR < 0.05 were identified and are listed in Supplementary Material S4. Among them, a forked transcription factor FOXO4 and its downstream targeting genes *RIT1*, *CLC*, *FKBP5*, *AGR2*, *CD36*, *RUNX2*, *NTRK2*, *GRK5*, *GATA2*, *ST6GAL1*, *SLC7A1*, *SLC18A2*, and *SERPINB10* were enriched. The previous studies showed that FOXOs were involved in multiple cellular processes, such as stress resistance, cell cycle, apoptosis, and metabolism [29]. In asthmatic patients, the increased expression of *AGR2* and *RUNX2* would lead to excessive secretion of mucin [30,31]. *GATA2* positively regulated the expression of IL-33 receptor (ST2), which stimulated the production of several pro-inflammatory factors in mast cells [32]. Furthermore, the increased expression of *IL-13* promoted the sialylation of MUC4β N-glycans by *ST6GAL1* and inhibited the proliferation of damaged epithelial cells in asthmatic patients [33]. The damaged epithelial cells can produce fibroblast-promoting growth factors, which aggravated the airway remodeling response [34].

Similarly, 24 potential regulatory microRNAs were identified (FDR < 0.05) (Supplementary Material S5). A previous study showed that the expression level of *microRNA-181a* was higher in the acute asthma patients compared to the healthy controls at the beginning of asthma, then dropped to the control level when there were no new airway stimuli. Therefore, *microRNA-181a* is a potential pro-inflammatory factor in asthma [35]. Mohamed et al. reported that the overexpression of *microRNA-26a* would increase hypertrophy of human airway smooth muscle cells and promote airway remodeling by inhibition of GSK-3β [36].

#### 2.4.4. Effect of Chromosomal Position on the Expression of DEGs

**Figure 5:**
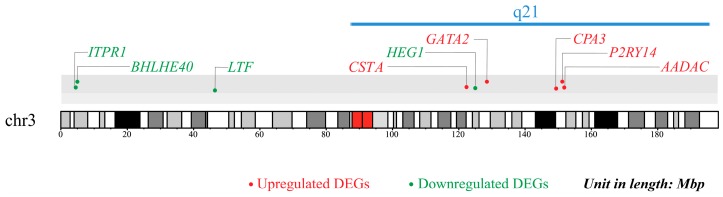
Chromosome localization of nine DEGs potentially associated with asthma

For many chromosomal loci, gene mutation and dysregulation of gene expression often result in many diseases including prostate cancer [37] and breast cancer [38]. Genome-wide linkage analysis of asthma and airway responsiveness showed that chromosome 12q24.31 contained a locus that was crucial in intermediate phenotype for asthma in a Hispanic population of Costa Rica [39]. Subsequently, Ferreira et al. found that chromosome 11q13.5 locus was significantly associated with the risk of allergic sensitization, in turn, increasing the risk of subsequent development of asthma in Australia [40]. In this study, gene position enrichment analysis of 153 DEGs revealed that 9 asthma-related genes were located on chromosome 3 (Figure 5). Among them, 5 up-regulated genes (*CSTA*, *GATA2*, *CPA3*, *P2RY14*, and *AADAC*) and 1 down-regulated gene (*HEG1*) were located in q21 region (FDR = 9.65 × 10^−5^), while the remaining 3 down-regulated genes (*LTF*, *ITPR1*, *BHLHE40*) were located out of q21 region on chromosome 3. Our results indicated that chr3q21 was a new locus associated with asthma.

### 2.5. Identification of Hub Genes Based on PPI Network Construction

**Figure 6:**
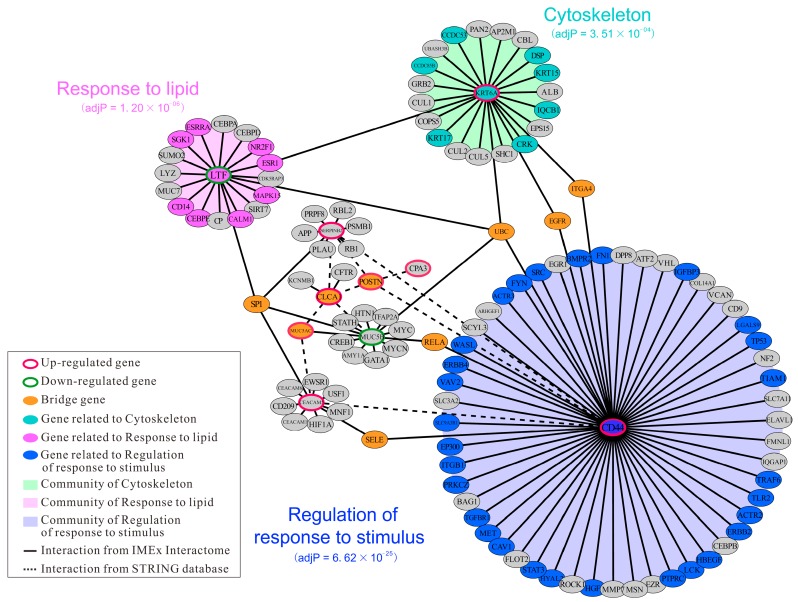
Protein-protein interaction network associated with asthma was constructed based on the DEGs with fold change > 1.5. The up- and down-regulated DEGs are marked in red and green circles, respectively. The solid lines and dashed lines represent the interactions predicted based on the IMEx interactome database and STRING database (v11.0), respectively. According to the degree of each node, six proteins with degree ≥ 10 were regarded as the hub proteins, and their corresponding genes were thought to be the hub genes, including *CD44* (degree = 59), *KRT6A* (degree = 23), *LTF* (degree = 18), *MUC5B* (degree = 13), *CEACAM5* (degree = 11), and *SERPINB2* (degree = 10).

To better understand the mechanism of asthma and identify the crucial hub genes among the DEGs, PPI network was constructed using network analysis, which enables the analysis of protein-protein interaction for multiple genes using NetworkAnalyst [41]. To categorize gene functions, genes in this network were divided into three groups: hub genes highly interacting with other neighbor genes (*CD44*, *KRT6A*, *LTF*, *SERPINB2*, *MUC5B*, and *CEACAM5*), bridge genes connecting different communities (e.g., *MUC5AC*, *CLCA1*, *POSTN*, *SP1*, and *EGFR*) and leaf-node genes with one neighbor (e.g., *CPA3*, *BAG1*, *MNF1*, and *APP*), as shown in Figure 6.

Within this network, CD44 encoding a cell surface adhesion receptor was the most highly ranked hub gene (degree = 59). A previous study showed that CD44 was significantly up-regulated in the bronchial epithelium in asthmatics compared with the controls [42]. Higher expression of CD44 in blood eosinophils could regulate the recruitment and function of leukocytes in asthmatics and are considered as a marker of bronchial asthma [43]. KRT6A are a member of type II epithelial keratins, which are intermediate filament-forming proteins that provide mechanical support and fulfil a variety of additional functions in epithelial cells. The presence of KRT6A was associated with pachyonychia congenital [44], as well as renal carcinoma [45] and breast cancer progression [46]. Genome-wide expression profiling with linkage analysis revealed that the transcription level of KRT6A significantly increased in peripheral blood mononuclear cells (PBMCs), airway brushing cells (ABCs), and bronchioalveolar lavage (BAL) when asthmatics were exposed to cockroach allergen [47]. In line with this, a recent proteomic study of asthma also found that KRT6A was up-regulated in the blood of asthmatics [48]. Therefore, KRT6A was considered as an important marker for asthma. Lactotransferrin (LTF), also called lactoferrin, is an iron-binding glycoprotein and servers as an immune-modulator and anti-inflammatory factor. Transcriptome analysis showed that LTF was significantly up-regulated during the development of asthma [49]. LTF could reduce pollen-induced airway inflammation in a mouse model of allergic asthma [50]. Immunoglobulin receptor CEACAM5, also known as CD66e, is a member of the carcinoembryonic antigen (CEA) family and involved in cell signaling, cell proliferation, cell repair responses, and the maintenance of the intact bronchial epithelium [51]. A previous study showed that the expression of CEACAM5 was increased in smoking and non-smoking severe asthma [52]. Consistent with this, the up-regulation of CEACAM5 was also observed in the epithelium in severe neutrophilic asthma [53].

Within bridge genes, MUC5AC is a glycoprotein and significantly increased when the respiratory tract was exposed to external stimulus [54]. High expression of MUC5AC was observed in bronchial epithelial cells of patients with severe asthma [55]. A recent study showed that IL-13 response genes (SERPINB2, POSTN, and CLCA1), protease genes (CPA3 and TPSAB1), and a nitric oxide synthase gene (NOS2) were significantly up-regulated in the epithelium of mild asthma [11].

To provide a comprehensive PPI network structure, the well-established network community recognition algorithm InfoMap [56] was applied and three distinct communities (marked with royal blue, dark turquoise or magenta areas) were obtained (*p* < 0.001). Combined with gene enrichment analysis, we found that they were associated with the regulation of response to stimulus, cytoskeleton, and response to lipid, respectively (Figure 6). This result suggested that the occurrence of asthma may be associated with the disturbance of lipid and abnormal cytoskeleton when epithelium cells were exposed to the stimulus.

### 2.6. Identification of Candidate Small Molecules

Using the 10 DEGs with fold change > 1.5 as seeds, 20 potential asthma-related chemical molecules with |score| > 0.6 and *p* < 0.05 were screened using a Connectivity Map (CMap)-based systems approach. Among them, 17 molecules probably promote the progression of asthma, such as Prestwick-1082, ricinine, and milrinone. On the contrary, 3 molecules have potential therapeutic effects, including catechin, lomefloxacin, and boldine (Table 5).

Catechins were one kind of polyphenols and showed various biological and pharmacological activities in antioxidative [57], anti-carcinogenic [58], and antiallergic effects [59]. Catechins from green tea could significantly inhibit the migration of inflammatory cells by suppressing MMP-9 expression and ROS generation in endothelial cells in a murine model of asthma [60]. Recently, Patel et al. confirmed that catechins from Acacia catechu showed inhibitory effects on ovalbumin-induced allergic asthma by inhibiting the activity of the histidine decarboxylase enzyme, and the author suggested that catechin can be used for the treatment of allergic inflammatory disease in humans [61].

Grassi et al. found that antibiotic lomefloxacin can treat acute exacerbations of chronic bronchitis [62]. Using the signalome screening method, Todd et al. reported that lomefloxacin showed significant effects on multiple pro-inflammatory signaling pathways that are constitutively activated in the auto-inflammatory disease TNF receptor [63]. However, no study has been performed concerning the usage of lomefloxacin for asthma treatment.

Boldine is a natural alkaloid from the leaves and bark of the Chilean boldo tree, *Peumus boldus*, and had anti-inflammatory [64] and antioxidant properties [65]. However, potential therapeutic effects on asthma have not been reported.

Taken together, the compounds catechin, lomefloxacin, and boldine may be potential drugs for the treatment of asthma caused by inflammation and allergy. However, further experiments will be needed to confirm the meta-analysis results.

### 2.7. Crosstalk Pathway of Asthma

To further explore the molecular mechanism of disease pathogenesis, functional enrichment analysis based on the BIOCARTA, REACTOME, and Pathway Interaction Database was performed by using the ToppGene Suite with the threshold of BH-FDR < 0.05.

Six categories were enriched, including 1) genes encoding ECM and ECM-associated proteins (M5889); 2) ECM-affiliated proteins, ECM-regulators and secreted factors (M5885); 3) HIF-1α transcription factor network; 4) FOXA1 transcription factor network; 5) termination of O-glycan biosynthesis, and 6) interleukin-4 and 13 signaling (Table 6). Interestingly, two asthma-associated pathways (M5889 and M5885) were enriched from the BIOCARTA database. Changes observed during airway remodeling in chronic asthmatic patients include excessive extracellular matrix (ECM) production and collagen deposition, increased airway smooth muscle mass, and mucus hypersecretion [66]. Abnormal deposition of ECM protein and/or ECM-associated proteins causes airway stiffening and narrowing, and differences in ECM protein expression may represent a specific asthma phenotype [67]. Our results strongly support that the accumulation of ECMs is essential for the development and progression of airway remodeling in asthma. Some components of ECMs may be novel therapeutic targets in the treatment of airway diseases by suppressing airway remodeling and inflammation.

**Figure 7:**
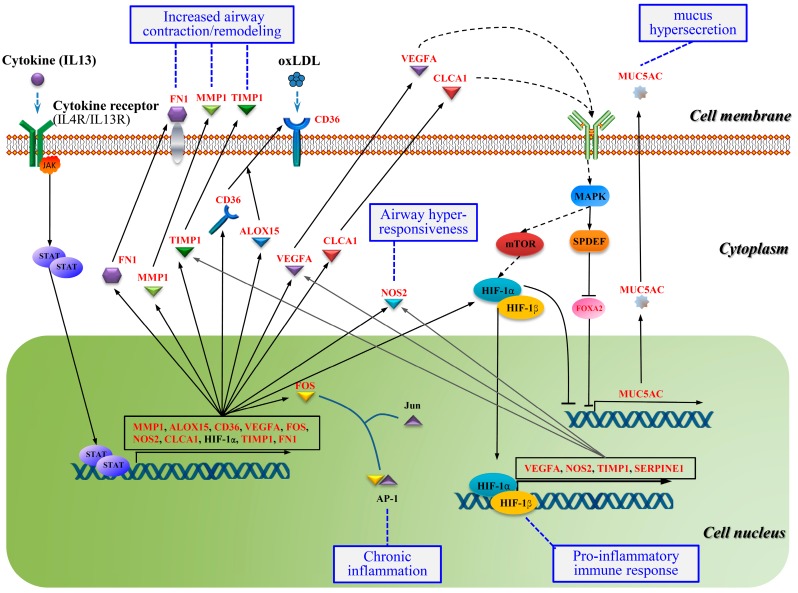
Cross-talking pathways associated with asthma inferred by DEGs as well as literature.

It is also worth noting that the networks driven by transcription factors HIF-1α and FOXA1 and the signaling mediated by the interleukin-4 and 13 (IL4 and IL13) receptors were enriched for asthma (Figure 7). Previous studies revealed that HIF-1α was a master regulator of inflammation and was up-regulated in the immune cells and lung tissues of asthma patients [68,69,70].

In addition, hypoxia-induced HIF-1α could increase the expression of *MUC5AC* via NF-kB-mediated signaling pathway in asthma. Knocking down *HIF-1α* by siRNA decreased *MUC5AC* expression under hypoxia even in IL-1β-treated cells. The above results suggest that HIF-1α may be a therapeutic target for asthma [71].

Cytokine receptors binding to IL-4 activate the JAK-STAT signaling pathway that regulates the expression of downstream genes *MMP1*, *ALOX15*, *CD36*, *VEGFA*, *FOS*, *NOS2*, *CLCA1*, *HIF-1α*, *TIMP1*, and *FN1* [72]. All target genes, except for *HIF-1α*, were identified in this study. Therefore, targeting this pathway through the inhibition of activating IL13 and IL4, and their receptors, and other pathway components should have therapeutic effects on asthma.

## 3. Materials and Methods

### 3.1. Microarray Gene Expression Data Acquisition

Publicly available microarray gene expression datasets for asthma were retrieved from the Gene Expression Omnibus Database (GEO) (http://www.ncbi.nlm.nih.gov/geo/) using the keyword “asthma”. The raw datasets were manually checked and only those that met the following criteria were included for subsequent analysis: 1) gene expression profiling in asthmatics and controls, 2) cell type: airway epithelial cell, but not nasal epithelium, 3) gene expression data were generated by a single-channel microarray platform (Affymetrix or Agilent chips), 4) availability of raw CEL or TXT files, and 5) samples with detailed descriptions.

### 3.2. Quality Control and Individual Microarray Dataset Preprocessing

Quality control (QC) analysis was performed for each microarray data using the arrayQualityMetrics package in R. Datasets were excluded from subsequent analysis when they failed to pass any one of the following assessments: 1) pairwise distances between arrays, 2) boxplots; 3) MA plots [73].

Briefly, the raw Affymetrix CEL files were normalized using the robust multi-array averaging (RMA) approach in the Affy package in Bioconductor Project with the following parameters: background correction, normalization and summarization [74], and returning log2 transformed intensities. Raw Agilent microarray data were normalized using the limma package, following background correction using the normexp method, quantile normalization and log2 transformation [75]. All analyses were performed with Bioconductor and R [76].

### 3.3. Data Integration and Batch Effect Removal

In this study, each dataset from all studies was preprocessed separately and then combined into one large dataset for further analysis following the steps illustrated in Figure 1. Probe identifiers from different microarray data were converted into Entrez gene IDs. If more than one probe mapped to a gene, the probe with the largest interquartile range (IQR) was selected as described by Letellier et al. [77], while a probe that mapped to multiple genes was excluded from further analysis.

The direct data integration (DDI) strategy presented in our previous publication [78] was applied to combine the multiple datasets in this study. Specifically, the shared genes among all microarray platforms used in this study were extracted, and then the multiple normalized datasets were merged into a large dataset (i.e., gene expression matrix) for shared genes.

To reduce potential study-specific batch effects, the preprocessed and normalized individual dataset was subjected to correction using the ComBat algorithm, which used the empirical Bayes method to adjust the extreme expression ratios and stabilized gene variances across all other genes and protected their reference from artifacts in the data [79]. The results of data integration and batch effect removal were visualized using clustering dendrograms, created by R and ggtree package [80].

### 3.4. Identification of Differentially Expressed Genes

Differentially expressed genes (DEGs) in integrated datasets were identified using limma package [75]. The linear fit, empirical Bayes (eBayes) statistics, and a false discovery rate (FDR) correction for all data were conducted using lmFit function. Genes with fold change > 1.2 and FDR < 0.05 were considered as DEGs between asthmatics and healthy controls. In addition, fold change > 1.5 and FDR < 0.05 were provided as well, hence we could identify genes which were more closely related to asthma.

### 3.5. Gene Ontology and Pathway Enrichment Analysis

To study the function of DEGs, enrichment of gene ontology (GO) was performed using DAVID (https://david.ncifcrf.gov/). The gene list was uploaded and analyzed using the annotation clustering for biological processes (BP), cellular components (CC), and molecular functions (MF). GO terms were considered to be significant when adjusted *p* < 0.01. The enrichment results were visualized using GOplot package [81]. Additionally, the enrichment analysis of Kyoto Encyclopedia of Genes and Genomes (KEGG)-based pathway was carried out using the GSEA online tool (http://www.broadinstitute.org/gsea/msigdb/index.jsp) [82]; KEGG pathways were considered to be significant when FDR < 0.05.

To identify up- or down-regulated terms based on DEGs, the Z-score proposed by Walter was adopted and calculated using the following formula [81]:

$$Z-score=\frac{N_{up}-N_{down}}{\sqrt{Count}}$$

where *N_up_*, *N_down_* represent the number of up- or down-regulated genes between asthmatics and healthy controls, respectively. The count is the number of DEGs belonging to this term.

To investigate the biological pathway most relevant to asthma, functional pathway enrichment analyses of DEGs were performed using ToppGene Suite [83].

### 3.6. Protein-Protein Interaction Network Construction and Community Detection

To explore potential protein-protein interaction associated with asthma, DEGs with fold change > 1.5 were mapped to the PPI data using NetworkAnalyst [41] and its built-in IMEx Interactome, as well as the STRING online database [84]. The PPI network was visualized using Cytoscape V3.5.1 [85].

The asthma-relevant hub-genes were screened using the node degrees calculated in Cytoscape, and the detection of communities in the PPI network was performed with the *InfoMap* algorithm [56].

### 3.7. Target Gene Prediction of Key Transcription Factors and Regulatory MicroRNAs

Analysis of key transcription factors and their putative target genes was carried out using the GSEA online tool [82]. The statistical significance was determined using hypergeometric distribution and followed by Benjamini-Hochberg multiple testing. Significantly enriched TFs and microRNAs were considered when FDR < 0.05.

### 3.8. Chromosome Position Effect on Gene Expression

To study whether chromosome position affected gene expression patterns, all DEGs were mapped and enriched on chromosomes using the GSEA online tool. The mapping results (FDR < 0.01) were visualized using the karyoploteR package [86].

### 3.9. Connection of DEGs and Small Chemical Molecules

The Connectivity Map was a database of gene expression patterns from cultured human cells treated with bioactive small chemical molecules, in which 6100 groups of small molecule interference experiments and 7056 corresponding gene-expression profiles were stored [87]. CMap analysis was helpful in identifying bioactive molecules resulting in similar or adverse gene expression patterns. DEGs with fold change > 1.5 were subjected to connective mapping analysis using the PharmacoGx package [88], and the enrichment score (ES) was calculated. A molecule was considered beneficial for asthma when ES < −0.5, while it was harmful when ES > 0.5.

## 4. Conclusions

To investigate the characteristics of gene expression profiles of bronchial epithelium from asthmatic patients, data integration and systematic bioinformatics approaches were applied in this study. A total of 78 significantly up-regulated and 75 down-regulated genes were identified. Further analyses revealed that 10 differentially expressed genes are possibly instrumental in asthma, including up-regulated *CEACAM5*, *CLCA1*, *POSTN*, *CPA3*, *SERPINB2*, *KRT6A*, *CD44*, *MUC5AC*, and down-regulated *LTF* and *MUC5B*. Especially, the present study suggests that *CD44*, *KRT6A*, *LTF*, *SERPINB2*, *MUC5B*, and *CEACAM5* could serve as hub genes in asthma-relevant PPI network and could further act as the biomarkers of asthma. Furthermore, the cross-talking of the IL-4/13 signaling pathway and networks intermediated by transcription factor HIF-1α and FOXA1 play a crucial role in the pathogenesis of asthma. Drug candidate prediction showed that compounds catechin, lomefloxacin, and boldine may be potential drugs for the treatment of asthma caused by inflammation and allergy. Our results elucidated the possible pathogenesis mechanisms of bronchial asthma and also provided several targets for further investigation.

## Figures and Tables

**Table 1:** Information summary of microarray datasets used in this study.

GEO ID	Microarray Platform	Sample Size (A/H) ^1^	Cell Type	Author (Year)
GSE470	HG-U95Av2	12 (6/6)	Epithelial cells	Spannhake W, et al. (2003)
GSE4302	HG-U133_Plus_2	70 (42/28)	Airway epithelial cells	Woodruff PG, et al. (2007) [8]
GSE18965	HG-U133A	16 (9/7)	Bronchial epithelial cells	Beyer RP, et al. (2010) [9]
GSE41861	HG-U133_Plus_2	81 (51/30)	Bronchial epithelial cells	Cheng DT, et al. (2015)
GSE44037	HT_HG-U133_Plus_PM	12 (6/6)	Bronchial epithelia	Wagener AH, et al. (2013) [14]
GSE63142	GPL6480 (Agilent)	155 (128/27)	Bronchial epithelia	Wenzel S, et al. (2014) [10]
GSE64913	HG-U133_Plus_2	59 (22/37)	Peripheral airway epithelia	Singhania A, et al. (2017) [15]
GSE67472	HG-U133_Plus_2	105 (62/43)	Airway epithelia	Christenson SA, et al. (2015) [16]
GSE89809	HT_HG-U133_Plus_PM	56 (38/18)	Epithelial cells	Singhania A, et al. (2017) [11]
GSE104468	GPL21185 (Agilent)	24 (12/12)	Bronchial epithelia	Richards A, et al. (2017) [17]

**^1^** A and H represent asthmatics and healthy controls, respectively.

**Table 2:** The significant DEGs with fold change > 1.5 between asthmatics and healthy controls

Gene	Entrez ID	Log2(Fold Change)	Asthmatics *vs.* Healthy Controls	FDR ^1^
*CEACAM5*	1048	1.13	Up	7.67 × 10^−23^
*CLCA1*	1179	1.58	Up	5.43 × 10^−22^
*POSTN*	10631	1.33	Up	7.83 × 10^−22^
*CPA3*	1359	1.26	Up	1.28 × 10^−21^
*SERPINB2*	5055	1.14	Up	9.55 × 10^−20^
*LTF*	4057	−0.75	Down	4.60 × 10^−17^
*MUC5B*	727897	−0.89	Down	2.10 × 10^−13^
*KRT6A*	3853	0.61	Up	4.89 × 10^−12^
*CD44*	960	0.60	Up	9.03 × 10^−9^
*MUC5AC*	4586	0.59	Up	1.03 × 10^−6^

**^1^** FDR (false discovery rate) refers to the BH-adjusted *p*-value returned by eBayes function in limma package.

**Table 3:** GO (gene ontology) term enrichment of DEGs between the asthmatics and healthy controls.

GO Term	Description	Count ^1^	Z-score	Adj. *p* ^2^	Category
GO:0042221	Response to chemical stimulus	34	−0.34	2.64 × 10^−4^	BP
GO:0032501	Multicellular organismal process	69	0.12	1.74 × 10^−3^	BP
GO:0018149	Peptide cross-linking	6	0.00	2.50 × 10^−3^	BP
GO:0005576	Extracellular region	51	0.42	1.67 × 10^−8^	CC
GO:0044421	Extracellular region part	31	0.18	4.21 × 10^−7^	CC
GO:0005615	Extracellular space	21	−0.65	4.33 × 10^−4^	CC
GO:0030141	Secretory granule	10	0.63	2.75 × 10^−3^	CC
GO:0031012	Extracellular matrix	13	0.28	4.33 × 10^−3^	CC
GO:0000267	Cell fraction	24	−0.82	6.39 × 10^−3^	CC
GO:0005624	Membrane fraction	20	−0.89	6.10 × 10^−3^	CC
GO:0005578	Proteinaceous extracellular matrix	12	0.00	5.90 × 10^−3^	CC
GO:0005626	Insoluble fraction	20	−0.89	7.52 × 10^−3^	CC

**^1^** Count denotes the number of DEGs in this GO term; **^2^** Adj. *p* refers to the adjusted *p*-values computed by DAVID.

**Table 4:** Enriched KEGG (Kyoto Encyclopedia of Genes and Genomes) pathways for DEGs using Molecular Signatures Database from GSEA (Gene Set Enrichment Analysis) online tool.

KEGG Pathway	FDR ^1^	Expression Pattern ^2^	Z-score	Gene
(1) Pathways in cancer	3.24 × 10^−5^	3↑+ 7↓	−1.26	*NOS2*, *MMP1*, *VEGFA*, *DAPK1*, *FOS*, *KIT*, *FN1*, *RUNX1T1*, *EPAS1*, *AR*
(2) Arachidonic acid metabolism	1.42 × 10^−4^	3↑+ 2↓	0.45	*CYP2J2*, *ALOX15*, *GPX3*, *HPGDS*, *PTGS1*
(3) Linoleic acid metabolism	7.69 × 10^−3^	2↑+ 1↓	0.58	*CYP2J2*, *ALOX15*, *AKR1B10*
(4) Calcium signaling pathway	1.17 × 10^−2^	1↑+ 4↓	−1.34	*NOS2*, *ITPR1*, *AVPR1A*, *PTGFR*, *TRPC1*
(5) Aldosterone-regulated sodium reabsorption	1.17 × 10^−2^	0↑+ 3↓	−1.73	*IRS2*, *INSR*, *SCNN1G*
(6) Bladder cancer	1.17 × 10^−2^	1↑+ 2↓	−0.58	*MMP1*, *VEGFA*, *DAPK1*
(7) Arginine and proline metabolism	1.95 × 10^−2^	2↑+ 1↓	0.58	*NOS2*, *ODC1*, *PYCR1*
(8) PPAR signaling pathway	3.34 × 10^−2^	2↑+ 1↓	0.58	*MMP1*, *CD36*, *FABP6*
(9) Leishmania infection	3.39 × 10^−2^	1↑+ 2↓	−0.58	*NOS2*, *FOS*, *HLA-DQB1*
(10) Cytokine-cytokine receptor interaction	3.51 × 10^−2^	2↑+ 3↓	−0.45	*VEGFA*, *KIT*, *CXCL2*, *CXCL6*, *CSF2RB*
(11) ECM-receptor interaction	4.39 × 10^−2^	2↑+ 1↓	0.58	*FN1*, *CD36*, *CD44*
(12) Hematopoietic cell lineage	4.62 × 10^−2^	3↑+ 0↓	1.73	*KIT*, *CD36*, *CD44*

**^1^** FDR denotes the FDR q-value provided by GSEA online tool; **^2^** ↑ refers to the status of up-regulated DEGs, and ↓ refers to the status of down-regulated DEGs.

**Table 5:** List of small molecules enriched from CMap database.

Molecules	Enrichment Score	*p*-Value
Catechin	−0.7995	0.0066
Lomefloxacin	−0.7101	0.0072
Boldine	−0.6635	0.0250
Prestwick-1082	0.6944	0.0099
Ricinine	0.7297	0.0102
Milrinone	0.7751	0.0153
Econazole	0.7981	0.0170
Acetohexamide	0.6542	0.0181
Cefsulodin	0.7900	0.0192
Nifuroxazide	0.7567	0.0199
Alimemazine	0.7744	0.0289
Progesterone	0.7258	0.0309
Zoxazolamine	0.7206	0.0390
Colistin	0.6503	0.0404
Methapyrilene	0.7618	0.0427
Tiapride	0.6832	0.0436
Fluocinonide	0.7451	0.0466
Ganciclovir	0.7389	0.0466
Quinisocaine	0.6790	0.0479
Mexiletine	0.7615	0.0492

**Table 6:** The enriched pathways from BIOCARTA, REACTOME and Pathway Interaction Database.

Pathway ID	Name	Database	FDR	Count ^1^
M5889	Genes encoding ECM and ECM-associated proteins	BIOCARTA (v6.0)	1.40 × 10^−2^	26
M5885	ECM-affiliated proteins, regulators and secreted factors	BIOCARTA (v6.0)	2.42 × 10^−2^	20
1470923	Interleukin−4 and 13 signaling	REACTOME	1.40 × 10^−2^	8
138045	HIF−1-alpha transcription factor network	Pathway Interaction Database	2.15 × 10^−2^	6
137979	FOXA1 transcription factor network	Pathway Interaction Database	2.23 × 10^−2^	5
1268737	Termination of O-glycan biosynthesis	REACTOME	2.61 × 10^−2^	4

**^1^** Count denotes the number of DEGs in this term.

## Supplementary Materials

Supplementary materials can be found at https://www.mdpi.com/1422-0067/20/16/4037/s1.

## Abbreviations

GO	Gene Ontology
KEGG	Kyoto Encyclopedia of Genes and Genomes
DEGs	Differentially Expressed Genes
GSEA	Gene Set Enrichment Analysis
PPI	Protein-protein Interaction
CMap	Connectivity Map
ES	Enrichment Score
