# Supplementary material for: Consistent Biomarkers and Related Pathogenesis Underlying Asthma Revealed by Systems Biology Approach
Source: Int J Mol Sci. 2019 Aug 19;20(16):4037. doi: 10.3390/ijms20164037 (PMC6720652; doi:10.3390/ijms20164037)
Supplement: Supplementary file 1 [file ijms-20-04037-s001.zip › ijms-566768-supplementary-XML/Supplementary Material S4-The potential target sites of transcription factors from GSEA, for 78 up-regulated DEGs.pdf]

S4. The potential target sites of transcription factors from GSEA, for 78 up-regulated DEGs.

**TF binding site**	**Conserved motif in target**	**Count**	**P-value**	**FDR**	**Genes**
FOXO4_01	TTGTTT	13	4.13E-05	1.27E-02	*RIT1, CLC, FKBP5, AGR2, CD36, RUNX2, NTRK2, GRK5, GATA2, ST6GAL1, SLC7A1, SLC18A2, SERPINB10*
LEF1_Q2	CTTTGT_	12	1.18E-04	1.45E-02	*RIT1, CLC, FKBP5, GRK5, GATA2, ST6GAL1, GSN, AKAP12, FHL1, DPYSL3, KIT, SLC39A8*
MAZ_Q6	GGGAGGRR_	12	4.36E-04	3.35E-02	*CLC, SERPINB2, NTRK2, GRK5, POSTN, GSN, AKAP12, IGF2BP3, SNCA, CD44, FGFBP1, AGPS*
AP1_C	TGANTCA_	10	2.01E-05	1.24E-02	*RIT1, CLC, FKBP5, AGR2, S100A2, SERPINB2, SPRR1A, MMP1, SPRR1B, TFF1*
TATA_01	TATAAA_	10	6.79E-05	1.39E-02	*RIT1, S100A2, SERPINB2, SPRR1A, CD36, RUNX2, POSTN, DEFB1, NOS2, HIST1H2BK*
AP1FJ_Q2	-	5	9.69E-05	1.45E-02	*CLC, S100A2, NTRK2, GSN, PTPRH*
UNKNOWN	SMTTTTGT_	5	6.25E-04	4.27E-02	*FKBP5, NTRK2, GRK5, GATA2, ST6GAL1, SLC7A1, PTGS1*
GFI1_01	TGATTTRY_	5	1.49E-04	1.53E-02	*AGR2, CD36, IGF2BP3, SNCA, NMU*
NFE2_01	TGASTMAGC_	4	3.51E-04	3.08E-02	*RIT1, CLC, S100A2, CD44*
