# Supplementary material for: Consistent Biomarkers and Related Pathogenesis Underlying Asthma Revealed by Systems Biology Approach
Source: Int J Mol Sci. 2019 Aug 19;20(16):4037. doi: 10.3390/ijms20164037 (PMC6720652; doi:10.3390/ijms20164037)
Supplement: Supplementary file 1 [file ijms-20-04037-s001.zip › ijms-566768-supplementary-XML/Supplementary Material S5-The potential target sites of regulatory microRNAs from GSEA, for 75 down-regulated DEGs.pdf]

S5. The potential target sites of regulatory microRNAs from GSEA, for 75 down-regulated DEGs.

microRNA	Recognized Motif	Count	P-value	FDR
MIR181A_MIR181B_MIR181C_MIR181D	TGAATGT	8	1.30E-06	2.87E-04
MIR200B_MIR200C_MIR429	CAGTATT	7	1.20E-05	1.33E-03
MIR15A_MIR16_MIR15B_MIR195_MIR424_MIR497	TGCTGCT	7	5.81E-05	4.28E-03
MIR19A_MIR19B	TTTGCAC	6	2.04E-04	8.74E-03
MIR506	GTGCCTT	6	1.23E-03	1.79E-02
MIR218	AAGCACA	5	4.99E-04	1.25E-02
MIR27A_MIR27B	ACTGTGA	5	1.09E-03	1.79E-02
MIR199A	CTACTGT	4	2.37E-04	8.74E-03
MIR144	ATACTGT	4	3.26E-04	1.03E-02
MIR204_MIR211	AAAGGGA	4	5.09E-04	1.25E-02
MIR320	CAGCTTT	4	8.38E-04	1.79E-02
MIR186	ATTCTTT	4	1.05E-03	1.79E-02
MIR374	TATTATA	4	1.23E-03	1.79E-02
MIR1425P	ACTTTAT	4	1.29E-03	1.79E-02
MIR1_MIR206	ACATTCC	4	1.50E-03	1.85E-02
MIR26A_MIR26B	TACTTGA	4	1.50E-03	1.85E-02
MIR520D	TTTGTAG	4	2.27E-03	2.64E-02
MIR383	TCTGATC	3	7.74E-05	4.28E-03
MIR21	ATAAGCT	3	9.27E-04	1.79E-02
MIR377	TGTGTGA	3	4.36E-03	4.34E-02
MIR511	AAAGACA	3	4.48E-03	4.34E-02
MIR448	ATATGCA	3	5.12E-03	4.71E-02
MIR2993P	CCCACAT	2	3.56E-03	3.93E-02
MIR514	GTGTCAA	2	4.52E-03	4.34E-02
