# Supplementary figures and images for: Consistent Biomarkers and Related Pathogenesis Underlying Asthma Revealed by Systems Biology Approach
Source: Int J Mol Sci. 2019 Aug 19;20(16):4037. doi: 10.3390/ijms20164037 (PMC6720652; doi:10.3390/ijms20164037)

**HG-U133\_Plus\_2**

**HG-U95Av2**

**HG-U133A**

**GPL6480 (Agilent)**

**HT\_HG-  
U133\_Plus\_PM**

**GPL21185 (Agilent)**

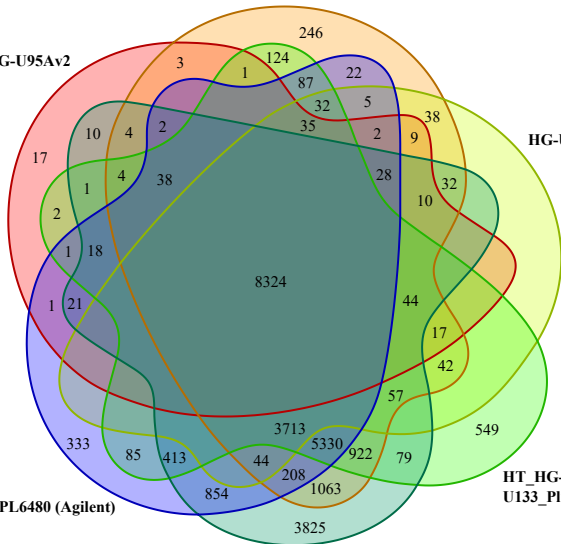

Supplement: Supplementary file 1 [file ijms-20-04037-s001.zip › ijms-566768-supplementary-XML/Supplementary Figure-S1-the shared genes among six platforms.pdf]
